# A depression network caused by brain tumours

**DOI:** 10.1007/s00429-022-02573-z

**Published:** 2022-10-03

**Authors:** Yanran Li, Yong Jin, Di Wu, Lifang Zhang

**Affiliations:** 1 Department of Radiology, First Affiliated Hospital of Xinjiang Medical University, Urumqi, 830054 China; 2 Department of Radiology, Changzhi People’s Hospital, Changzhi, 046000 Shanxi Province China; 3 Department of Neurology, Xuanwu Hospital, Capital Medical University, Beijing, 100053 China; 4 Department of Neurology, Changzhi People’s Hospital, No. 502 of Changxing Middle Street, Luzhou District, Changzhi, 046000 Shanxi Province China

**Keywords:** Brain tumour, Depression, Brain network, Lesion-network mapping

## Abstract

To systematically analyse and discuss whether suppressive heterogeneous brain tumours (BTs) belong to a common brain network and provide a theoretical basis for identifying BT patients with a high risk of depression and select therapeutic targets for clinical treatment. The PubMed database was systematically searched to obtain relevant case reports, and lesion locations were manually traced to standardised brain templates according to ITK-SNAP descriptive literature. Resting-state functional magnetic resonance imaging data sets were collected from 1,000 healthy adults aged 18–35 years. Each lesion location or functional connectivity area of the lesion network. Connectivity analysis was performed in an MN152 space, and Fisher *z*-transformation was applied to normalise the distribution of each value in the functional connectivity correlation map, and *T* maps of each tumour location network were calculated with the *T* score of individual voxels. This *T* score indicates the statistical significance of voxelwise connectivity at each tumour location. The lesion networks were thresholded at *T* = 7, creating binarised maps of brain regions connecting tumour locations, overlaying network maps to identify tumour-sensitive hubs and also assessing specific hubs with other conditional controls. A total of 18 patients describing depression following focal BTs were included. Of these cases, it was reported that depression-related tumours were unevenly distributed in the brain: 89% (16/18) were positively correlated with the left striatum, and the peak of the left striatum lesion network continuously overlapped. The depression-related tumour location was consistent with the tumour suppressor network (89%). These results suggest that sensitive hubs are aligned with specific networks, and specific hubs are aligned with sensitive networks. Brain tumour-related depression differs from acute lesion-related depression and may be related to the mapping of tumours to depression-related brain networks. It can provide an observational basis for the neuroanatomical basis of BT-related depression and a theoretical basis for identifying patients with BTs at high risk of depression and their subsequent clinical diagnosis and treatment.

**Supplementary Information:**

The online version contains supplementary material available at 10.1007/s00429-022-02573-z.

## Introduction

Primary brain tumours (BTs) have a worldwide incidence of approximately 7 per 1,000,000 (Parkin et al. 2002). Brain tumours currently require multimodal therapy. A quarter of patients with glioblastoma multiforme survive beyond 2 years after diagnosis, and other patients with malignant primary BT usually have a poorer prognosis (Stupp et al. 2005). Progressive neurological damage caused by BT invasions or side effects of treatment can lead to progressive deterioration of the condition. Patient-centred, including symptoms of psychological distress and side effects of treatment, are increasingly used as secondary indicators of treatment efficacy in clinical trials and should be considered when guiding individualised treatment for patients (Basch 2013; Bruner et al. 2007). Therefore, it is very important to accurately identify BT patients with mental illnesses in clinical practice. Assessments of psychological distress are also a priority for recent psycho-oncology research (Rankin et al. 2011). Depression is a common complication in cancer patients. Patients with depression are not easily identified and are often initially left untreated. Understanding the relationship between BTs and depression will help to improve the care and quality of life for patients with BTs.

The association between the location of brain lesions and depression is extremely complex. The cause of neuropsychiatric symptoms from neuroanatomy can be investigated in patients with focal brain lesions (Karnath et al. 2018; Rorden et al. 2007). Associations between left frontal lobe disease and depression have been found in stroke (Robinson et al. 1984) and BT patients (Wellisch et al. 2002; Belyi 1987). Further studies have found that depression is associated with bilateral dorsolateral prefrontal cortex (DLPFC) lesions (Koenigs et al. 2008), and DLPFC lesions span several Brodmann areas (BAs), including the dorsal portions of BAs 9, 8a, 8b and 46 (Sallet et al. 2013). However, there are differing views on the location of depression associated with focal brain damage. It has been found that the lesion site in patients with post-stroke depression is located outside the left frontal cortex (Robinson et al. 1984; Robinson and Price 1982). Moreover, some studies attempting to validate this association have found that it does not exist or only appears at specific timepoints (Kutlubaev and Hackett 2014). This association has not been found in many evidence-based medical studies and in new voxel-based mappings of lesion symptoms (Wei et al. 2015; Ayerbe et al. 2013; Yu et al. 2004; Gozzi et al. 2014; Sagnier et al. 2019).

From a functional perspective, increasing evidence suggests that brain lesions causing specific neurological symptoms are more likely to map to brain function networks rather than brain regions. A recently developed technique, called lesion-network mapping, can noninvasively identify intrinsic connections of different brain lesions causing specific symptoms and test whether they map to a common brain network. Using a database of standard resting-state functional connectivity data obtained from an appropriately large number of healthy subjects (*n* = 1000), also called standard connectivity groups, lesion-network mapping revealed networks associated with a set of neuropsychiatric symptoms.

This novel approach has been successfully used in depression. Lesion locations associated with depression are surrounded by brain circuits defined by the DLPFC that are significantly and specifically associated with depression (Padmanabhan et al. 2019). This depression circuit is derived from lesions of multiple etiologies, including cerebral haemorrhage, penetrating traumatic brain injury and ischemic stroke. However, these causes are acute, and the lesions occur almost simultaneously with the occurrence of depression, the cause of which itself can be regarded as simply due to the lesion. However, depression in patients with comorbid cancer depression may be associated with both the location of the tumour itself and long-term psychological or social stress, so lesion mapping network technology is required to explore whether tumour-related depression can be localised in a functional network-like depression due to acute injury. This network mapping determines the therapeutic target to relieve or prevent symptoms on the basis of a deep understanding of the neuroanatomy of tumour-induced neuropsychiatric symptoms (National Comprehensive Cancer Network 2003). Therefore, according to the specific symptoms of patients with BTs, this study identified the internal relations of different brain lesions causing depression through the technology of lesion-network mapping and tested whether they can be mapped to a common brain network, such as the depression network defined by acute lesions. Through this network mapping of tumour locations, it is anticipated that therapeutic targets can be identified that can alleviate or prevent symptoms based on an in-depth understanding of the neuroanatomy of neuropsychiatric symptoms caused by tumours.

## Materials and methods

### Case selection

Case reports describing patients with depression following focal BTs were systematically reviewed from the published literature. We searched PubMed for articles describing human subjects written in English with the terms: [(depression) or (suicide)] and (brain tumor) according to the preferred reporting items guide for systematic reviews and meta-analyses (Moher D et al. 2009). The inclusion criteria were (1) a clear description of depression over a long period, (2) depression after a brain tumour was identified, and (3) a published figure that showed the location of the tumour. The exclusion criteria were (1) patients with depression caused by but not limited to focal brain tumour, (2) the tumour location could not be reliably localised because of poor image quality, and (3) depression still existing after tumour resection.

### Tumour locations

Tumour locations, displayed in the original publications, were manually traced onto a standardised brain template (0.5 × 0.5 × 0.5 mm, MNI152 2009b) based on neuroanatomical landmarks using ITK-SNAP (www.itksnap.org) software. It is of note that this approach, which approximated the 3-dimensional (3D) lesions, has been consistently validated in its sufficiency for lesion-network mapping by prior works (Boes et al. 2015; Darby et al. 2017).

### Lesion-network mapping

To identify the network of brain regions functionally connected to each tumour location, connectivity analysis in an MNI152 space at 2 × 2 × 2 mm voxel size resolution was performed using resting-state functional magnetic resonance imaging data sets collected from 1,000 healthy adult subjects (Yeo et al. 2011). Fisher *z*-transformation was applied to normalise the distribution of values for each of the 1,000 functional connectivity-related graphs. *T* maps were computed for each lesion network with T-score values for each voxel. This *T* score represents the statistical significance of the connection of each voxel to the lesion location. Each lesion network was subsequently thresholded at *T* = 7 to create a binarised map of brain regions connected to the lesion location (corresponding to a voxelwise overall error-corrected *P* < 10^–6^). This threshold is consistent with previous publications (Kim et al. 2019; Joutsa et al. 2018).

### Sensitivity testing

To test for sensitivity, each lesion network was subsequently thresholded at *T* = 7 to create a binarised map (corresponding to voxelwise familywise error [FWE]-corrected *P* < 10^–6^) of brain regions connected to the lesion location. This threshold is consistent with previous publications. To ensure that our results did not depend on *T* thresholds, we repeated our analysis using *T* thresholds of 5 (voxelwise FWE-corrected *P* < 0.05), 9 (voxelwise FWE-corrected *P* < 10^–9^) and 11 (voxelwise FWE-corrected *P* < 10^–11^). Finally, all binarised maps for each lesion were overlapped to identify regions with shared connectivity and to treat this region as a sensitive hub.

### Specificity testing

To test for specificity, the unthresholded lesion-network map of BT-induced depression was compared with the unthresholded lesion-network map of control lesions of other BT-induced neurological syndromes (*n* = 72) through a two-sample *T* test (voxelwise FWE-corrected *P* < 0.05). These lesions were selected because they are the lesions causing neurological symptoms that I currently sketch and get connectivity function. Control lesions were consistent with lesions of different etiologies, including haemorrhages and tumours. In control lesions, 17 lesions causing amnesia (Ferguson et al. 2019) and 12 lesions causing Parkinson’s disease were selected from prior works using the lesion-network mapping technique (Joutsa et al. 2018), and 20 lesions causing facial palsy and 23 lesions causing vertigo were selected from PubMed.

### Defining a depression network of brain tumour

By definition, the connectivity with positive hubs identified by the combined analysis of sensitivity and specificity tests defined a distributed brain network that included tumour locations that caused depression while avoiding control lesions.

### Relationship between sensitive hubs and specific hubs

The connectivity between the sensitive and specificity hubs was used to define distributed brain networks. To illustrate these networks, these two hubs were running as seeds through the same normative used for studying brain lesions and thresholded at *T* =  ± 7 (corresponding to voxelwise FWE-corrected *P* < 10^–6^). The sensitive hub was overlaid on specificity hub networks, and the specificity hub was overlaid on sensitive hub networks to illustrate the relationship between these two hubs.

## Results

### Literature inclusion

A total of 2860 articles were retrieved, and 1655 were excluded after reviewing the article titles and abstracts. After reading the abstracts of the remaining articles, 1099 articles that did not meet the inclusion criteria were excluded. Eighty-eight full-text articles were excluded because they did not meet the exclusion criteria. Finally, case reports of 18 patients with tumour-related depression were included after reading the full text (Supplementary Figure 1).

### Tumours associated with depression are spatially heterogeneous

**Fig. 1:**
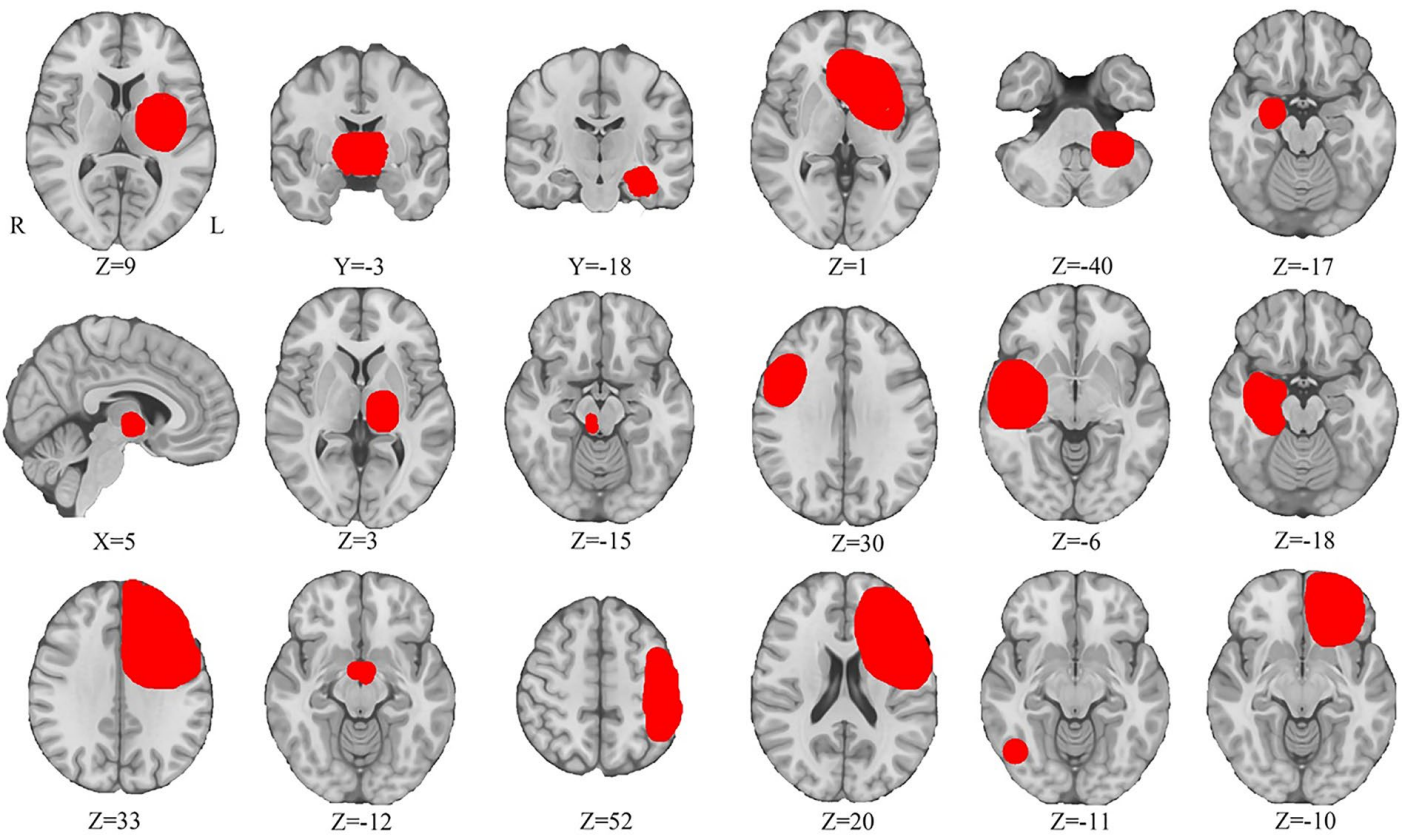
Tumor locations associated with depression. Tumor locations, numbered 1 through 18, were manually traced onto a standardized brain template (0.5 × 0.5 × 0.5 mm, MNI152 2009)

Eighteen patients with a documented relationship between BTs and depression were identified (Supplementary Table 1). Tumour locations were spatially heterogeneous (Fig. 1) and spanned multiple different brain regions, including the temporal lobe/hippocampus (*n* = 6), the frontal lobe (*n* = 3), bilateral thalami (*n* = 3), the mid-brain and diencephalic (*n* = 2), the parietal lobe (*n* = 1), bilateral thalami (*n* = 1), the insular (*n* = 1) and the cerebellopontine angle (*n* = 1).

### Lesion-network mapping of tumours associated with depression

**Fig. 2:**
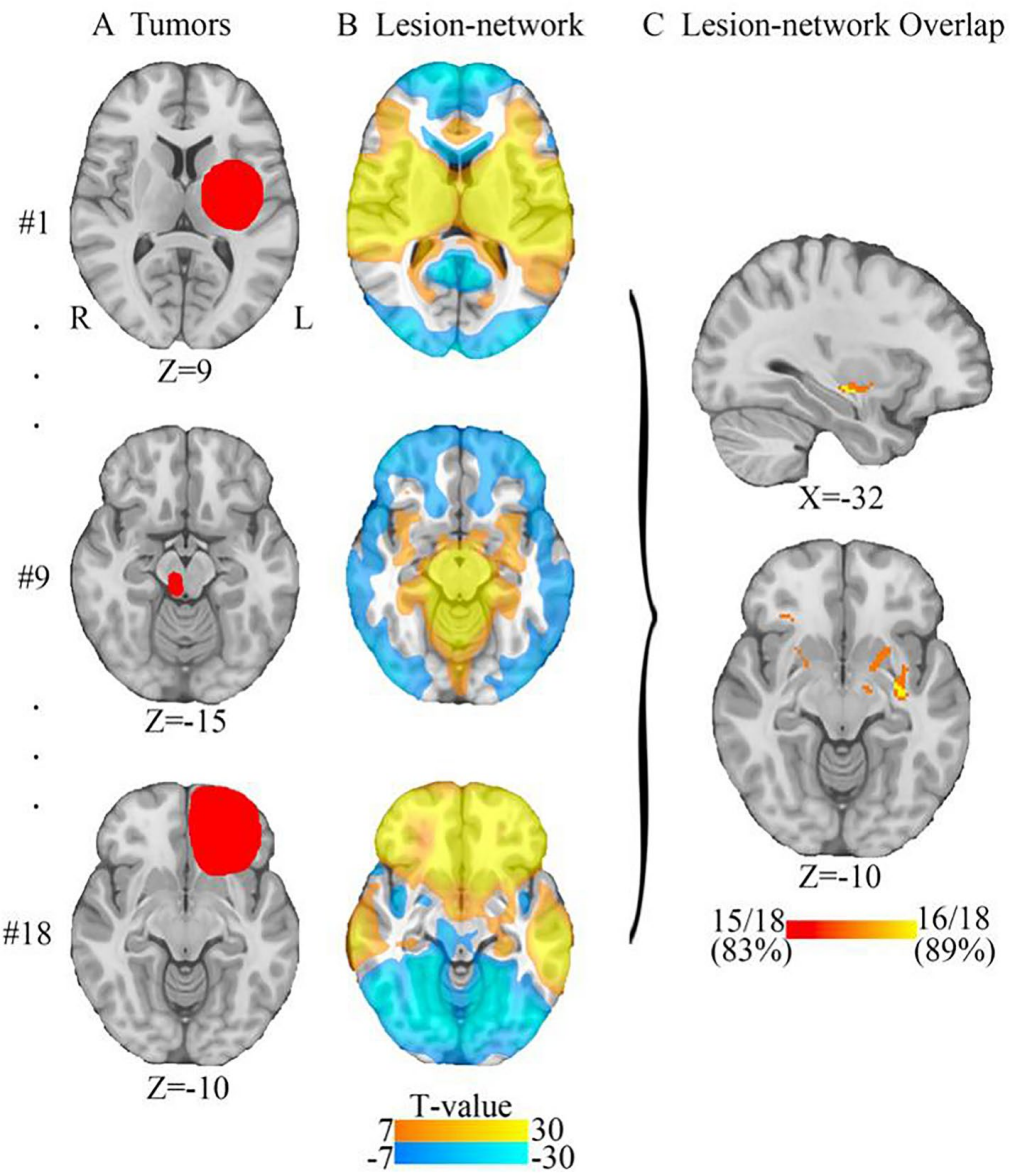
Lesion-network mapping of depression associated with tumor. **A** Representative tumor locations associated with depression. **B** Lesion-networks, representing regions functionally connected to tumor locations associated depression, were computed using a normative connectome (*n* = 1,000). **C** Lesion-network overlap (sensitivity testing) showing regions connected to most (16/18, 89%) tumor locations

While tumour locations associated with depression were heterogeneously distributed across the brain, 89% (16/18) of these tumour locations were positively connected to the left striatum with the *T* threshold at 7 (corresponding to voxelwise FWE-corrected *P* < 10^–6^) (Fig. 2). The peak lesion-network overlap in the left striatum were constant and independent of the *T* threshold of 5 (voxelwise FWE-corrected *P* < 0.05), 9 (voxelwise FWE-corrected *P* < 10^–9^) and 11 (voxelwise FWE-corrected *P* < 10^–11^) (Supplementary Figure 2).

### Depression network defined by sensitive hubs

**Fig. 3:**
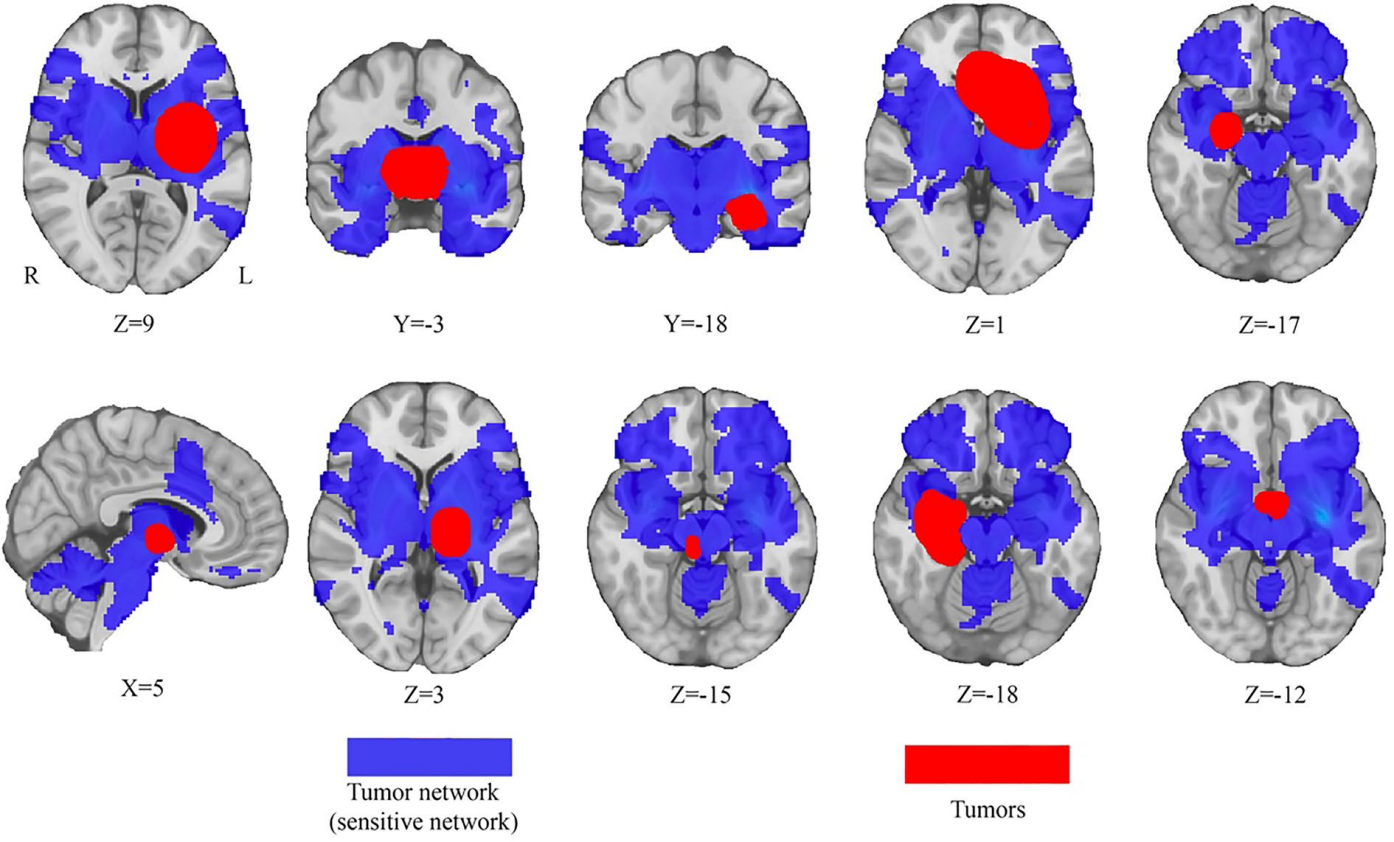
Tumor-based depression network. The sensitive test identifies the sensitive hub (the left striatum) of the tumor-based depression network (blue). This tumor-based depression network (blue) encompasses heterogenous tumor locations associated with depression (red)

**Fig. 4:**
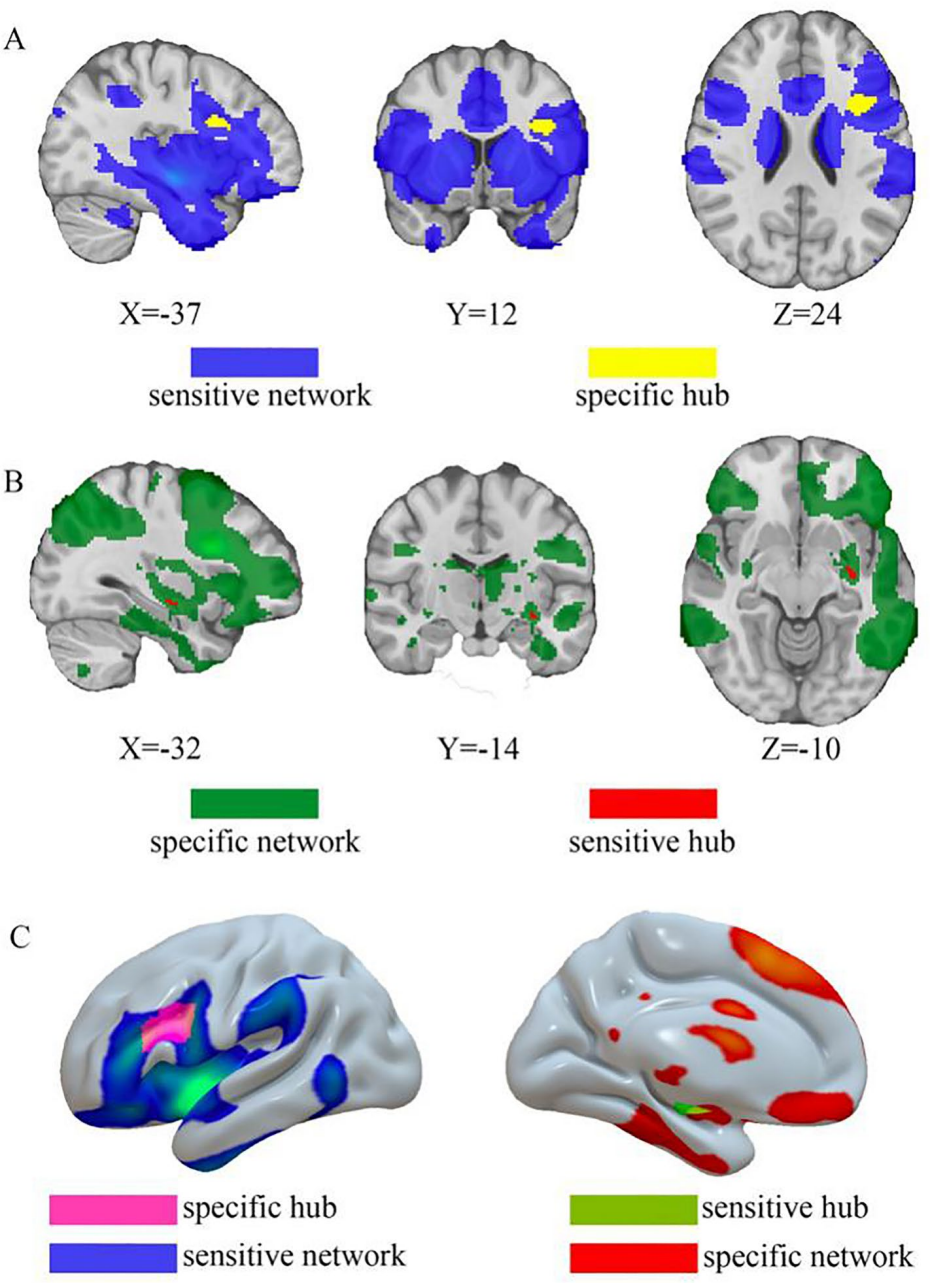
Relationship between sensitive hub and specific hub. **A** Sensitive network (blue) compasses the specific hub (yellow). **B** Specificity network (green) compasses the sensitive hub (red). **C** Relationship of 2 hubs and 2 networks in 3D vision

The network was defined by positive connectivity to the lesion-based hub in the left striatum (Fig. 2C). This network will be referred to as the tumour depression network of sensitive hubs (Fig. 3). This network is bilaterally organised, including the bilateral DLPFC, temporal lobe, insula cortex, hippocampus, cingulate cortex, precentral gyrus, superior marginal gyrus and thalamus. As expected, tumours associated with depression aligned well (89%) with the tumour depression network (Fig. 3). By the use of the sensitive test, we found the sensitive hub, which is shown in Figs. 2C, 4B, and 4C. This study’s depression network includes multiple subtentorial nodes, such as the vermis, bilateral dentate nucleus and cerebellum VIII. The striatum is the sensitive hub, and the left DLPFC is consistent with the sensitive network, as shown in Figs. 2, 3, and 4.

### Relationship between sensitive hubs and specific hubs

To test for specificity, we compared unthresholded lesion-network maps of depression with that of other symptoms (*n* = 72). The specific hub (FWE *P* < 0.05) for tumours causing depression was located in the left DLPFC (Fig. 4A). The specificity network was also calculated (Fig. 4B). The DLPFC is the specific hub, and the left striatum is consistent with the specificity network, which shows the intrinsic relationship between the striatum and DLPFC (Fig. 4).

## Discussion

Using a novel network mapping technique, this study found that neuroanatomically heterogeneous BTs in depression were located in a common brain network defined by the left striatum, involving the bilateral DLPFC, temporal lobe, insular cortex, hippocampus, cingulate cortex and thalamus. As expected, tumours associated with depression aligned well with the onco-depression network (89%). The depressed network comprised multiple infratentorial lymph nodes, including the vermis, bilateral dentate nuclei and cerebellum VIII.

In this study, the tumour location was spatially heterogeneous and spanned multiple distinct brain regions, including the temporal/hippocampal, frontal, bilateral thalamic, parietal, bilateral thalamic, insular, midbrain and diencephalon and cerebellopontine angle. Thus, we could not anatomically find a brain region surrounded by all tumour locations. However, we found that the left striatum was the most sensitive region defined for all tumour locations, and connectivity with the left striatum defined a human brain circuit that best contained tumour locations associated with depression. Based on BTs, this circuit may help refine the circuit model of depression. In contrast to the negative findings for lesion location, functional connectivity between tumour locations and the rest of the brain was a significant predictor of depression. This finding is consistent with a growing body of literature suggesting that symptoms are confined to connected brain circuits rather than individual brain injuries (Fox 2018). Therefore, this circuit may be used to identify an increased risk of depression in patients with BTs. Clinicians can examine the overlap between patient lesions and these tumour suppressor circuits. High-risk patients can guide future early psychiatric evaluation and treatment.

However, unlike previous studies using lesion-network mapping techniques, sensitive and specific hubs were not found to be in the same region. The depressed group showed higher functional connectivity between left DLPFC tumour locations and certain regions than the non-depressed group, even in the conservative whole-brain analysis with voxelwise FWE correction. This result is consistent with previous studies using network mapping of depressed lesions with focal brain injury, including ischemic stroke, cerebral haemorrhage and penetrating traumatic brain injury (Padmanabhan et al. 2019). The left DLPFC may have implications for understanding and treating primary depression caused by non-focal brain injury. Although depression is not necessarily associated with changes in the DLPFC activity (Kaiser et al. 2015; Zhong et al. 2016), increases in the DLPFC activity are associated with antidepressant response (Fitzgerald et al. 2006), particularly improvements in cognitive symptoms of depression.

This study’s results identify a relationship between the left striatum (sensitive centre) and the left DLPFC (specific centre), which is anatomically consistent with previously reported frontal white matter projections based on ex vivo nonhuman primate histology (Averbeck et al. 2014; Haber et al. 1995). Previous studies have shown that the striatum, thalamus and prefrontal cortex (PFC) constitute the prefrontal–subcortical circuit (Tekin and Cummings 2002), which is involved in emotional and cognitive processes (Marchand 2010) and is considered a potential pathophysiological target for depression.

Structural connectivity of the prefrontal–subcortical circuit begins in the PFC. The striatum receives information from the PFC and outputs it to the globus pallidus and substantia nigra. All information then projects through the thalamus to the PFC (Alexander et al. 1986). The thalamus is the final neuronal connection that returns to the cortex, making the circuit a closed loop. In the pathophysiological view, the PFC, hippocampus and thalamus elicit excitation and direct projections to the striatum. The striatum transmits information via GABAergic neurons projecting to the globus pallidus. Damage to this pathway may lead to pathological differences, and its injury leads to abnormal inhibition of the thalamus (Zhang et al. 2018). Kevin et al. (Jarbo and Verstynen 2015) found that although the distribution of cortical areas associated with striatal convergence areas differed somewhat between structural and functional connectivity measures, reflecting the methodological limitations of each method, most cortical areas structurally connected to convergence areas also showed strong functional connectivity. This supports this study’s findings that these corticostriatal projections are responsive to different symptoms associated with specific functional networks. According to this study’s results, the depression circuits derived from acute lesions and BT circuits are not entirely consistent. Thus, the intrinsic mechanisms of depression programmes in acute and chronic lesions differ. Depressive circuits caused by acute lesions can be explained by the lesion site itself, while depressive circuits caused by chronic lesions (e.g., tumours) cannot be explained by the tumour site itself or by psychological multifactorial unpleasant emotional experiences, i.e., social and/or psychiatric domains that may be associated with depressive procedures. This study’s tumour suppressor circuit is likely to have implications not only for understanding and treating primary depression caused by non-focal BTs but also for reminding us that patients reporting depressive symptoms should be considered high risk and referred for more detailed psychiatric evaluation.

This article has a certain novelty and superiority. First, the lesion-mapping network technology was used to explore whether there was the same brain network between depression caused by acute lesions and tumour-related depression. Based on the in-depth understanding of neuroanatomy of neuropsychiatric symptoms caused by tumours, the symptoms were alleviated or prevented by drawing the tumour location network, as the therapeutic target was determined. Second, through a strict search strategy, the eligible research reports were screened, which accurately reflected the brain network area of depression caused by BTs, but also distinguished depression caused by acute lesions.

This study has several limitations. First, as mentioned above (Darby et al. 2017), pathological network mapping using normative data sets assumes that healthy individuals’ connectivity patterns are nearly identical to those of patients before their brain lesions. This hypothesis is reasonable given the successful mapping of the lesion network across multiple symptoms (Boes et al. 2015; Darby et al. 2017; Horn et al. 2017; Fischer et al. 2016), and the preoperative use of age-matched connectors in diseased patients does not affect outcomes (Boes et al. 2015). Second, the accuracy of manual lesion tracking is limited by the quality of published images, and two-dimensional (2D) lesions were used based on published images, which may not fully capture the spatial extent of 3D lesions. However, previous studies showed that the connectivity of 2D representation of 3D lesions is highly similar to 3D lesions themselves (spatial correlation coefficient > 0.9) (Boes et al. 2015; Darby et al. 2017). Furthermore, any error in lesion tracking should lead to a tendency to find consistent network localisation between lesions. Third, depression is a syndrome composed of multiple different symptoms with potentially distinct neuroanatomical correlations. In the future, this technology needs to be applied to individual symptoms of depression.

## Conclusions

This study finds that the left striatum may be a brain function network associated with BTs and depression, which differs from the depression network defined by acute lesions. The above results are expected to provide an observation basis for the neuroanatomical basis of BT-related depression and provide a theoretical basis for identifying patients with BTs at high risk of depression and, subsequently, their clinical diagnosis and treatment.

## Supplementary Information

Below is the link to the electronic supplementary material.

Supplementary file1 (DOCX 561 KB)

## Data Availability

All data generated or analyzed during this study are included in this published article.
