# Supplementary material for: A depression network caused by brain tumours
Source: Brain Struct Funct. 2022 Oct 3;227(8):2787–95. doi: 10.1007/s00429-022-02573-z (PMC9618495; doi:10.1007/s00429-022-02573-z)
Supplement: Supplementary file 1 — Supplementary file1 (DOCX 561 KB) [file 429_2022_2573_MOESM1_ESM.docx]

**SUPPLEMENTARY TABLE 1**

**References for the 18 case studies meeting inclusion criteria.**

| Number | References for depression case studies meeting inclusion criteria |
| --- | --- |
| NO.1 | Bunevicius A, Deltuva VP, Deltuviene D, Tamasauskas A, Bunevicius R. Brain lesions manifesting as psychiatric disorders: eight cases. CNS Spectr. 2008 Nov;13(11):950-8. doi: 10.1017/s1092852900014000. PMID: 19037181. |
| NO.2 | Estronza S, Saavedra FM, De Jesus O, Pastrana EA. Chordoid Glioma with Psychosis: Case Report. P R Health Sci J. 2018 Sep;37(3):174-176. PMID: 30188563. |
| NO.3 | Betul O, Ipek M. Brain tumor presenting with psychiatric symptoms. J Neuropsychiatry Clin Neurosci. 2011 Fall;23(4):E43-4. doi: 10.1176/jnp.23.4.jnpe43. PMID: 22231349. |
| NO.4 | Tanaghow A, Lewis J, Jones GH. Anterior tumour of the corpus callosum with atypical depression. Br J Psychiatry. 1989 Dec;155:854-6. doi: 10.1192/bjp.155.6.854. PMID: 2620215. |
| NO.5 | Arifin MZ, Yudoyono F, Setiawan C, Sidabutar R, Sutiono AB, Faried A. Comprehensive management of frontal and cerebellar tumor patients with personality changes and suicidal tendencies. Surg Neurol Int. 2014 Dec 8;5:174. doi: 10.4103/2152-7806.146487. PMID: 25593758; PMCID: PMC4287917. |
| NO.6 | Lingeswaran A, Barathi D. Depression associated with dysembryoblastic neuroepithelial tumor. Indian J Psychol Med. 2012 Jul;34(3):273-5. doi: 10.4103/0253-7176.106028. PMID: 23439941; PMCID: PMC3573580. |
| NO.7 | Spence SA, Taylor DG, Hirsch SR. Depressive disorder due to craniopharyngioma. J R Soc Med. 1995 Nov;88(11):637-8. PMID: 8544149; PMCID: PMC1295388. |
| NO.8 | Lo Buono V, Corallo F, De Cola MC, Chillemi A, Grugno R, Bramanti P, Marino S. Effect of cognitive rehabilitation in a case of thalamic astrocytoma. Appl Neuropsychol Adult. 2016 Jul-Aug;23(4):309-12. doi: 10.1080/23279095.2015.1053888. Epub 2015 Nov 17. PMID: 26578385. |
| NO.9 | Ghaziuddin N, DeQuardo JR, Ghaziuddin M, King CA. Electroconvulsive treatment of a bipolar adolescent postcraniotomy for brain stem astrocytoma. J Child Adolesc Psychopharmacol. 1999;9(1):63-9. doi: 10.1089/cap.1999.9.63. PMID: 10357519. |
| NO.10 | Maurice-Williams RS, Sinar EJ. Depression caused by an intracranial meningioma relieved by leucotomy prior to diagnosis of the tumour. J Neurol Neurosurg Psychiatry. 1984 Aug;47(8):884-5. doi: 10.1136/jnnp.47.8.884. PMID: 6470732; PMCID: PMC1027958. |
| NO.11 | Franssen A, Sienaert P. Temporal glioblastoma presenting as catatonia. BMJ Case Rep. 2019 Mar 4;12(3):e224017. doi: 10.1136/bcr-2017-224017. PMID: 30837231; PMCID: PMC6424181. |
| NO.12 | Liao Y, He Y, Yang Y, Li X, Huang F. Case report: narcolepsy type 2 due to temporal lobe glioma. Medicine (Baltimore). 2020 Jul 10;99(28):e21002. doi: 10.1097/MD.0000000000021002. PMID: 32664104; PMCID: PMC7360319. |
| NO.13 | Petzold J, Severus E, Meyer S, Bauer M, Daubner D, Krex D, Juratli TA. Glioblastoma multiforme presenting as postpartum depression: a case report. J Med Case Rep. 2018 Dec 20;12(1):374. doi: 10.1186/s13256-018-1909-3. PMID: 30567605; PMCID: PMC6300872. |
| NO.14 | Gil-Simoes R, Pascual JM, Casas AP, de Sola RG. Intrachiasmatic craniopharyngioma: Assessment of visual outcome with optical coherence tomography after complete surgical removal. Surg Neurol Int. 2019 Jan 21;10:7. doi: 10.4103/sni.sni_292_18. PMID: 30775061; PMCID: PMC6357539. |
| NO.15 | Zivković N, Marković M, Mihajlović G, Jovanović M. Surgical treatment of intradiploic epidermoid cyst treated as depression. Srp Arh Celok Lek. 2014 Jan-Feb;142(1-2):67-71. doi: 10.2298/sarh1402067z. PMID: 24684034. |
| NO.16 | Ferreira LP, Pinheiro CF, Fernandes NA, Ferreira CN. When Depression Hides a Brain Tumor: A Case of Glioblastoma. Prim Care Companion CNS Disord. 2019 Nov 14;21(6):19l02455. doi: 10.4088/PCC.19l02455. PMID: 31730304. |
| NO.17 | Gonzalez A, Philpot MP. Late-onset startle syndrome and obsessive compulsive disorder. Behav Neurol. 1998;11(2):113-116. doi: 10.1155/1998/617160. PMID: 11568410. |
| NO.18 | Maruyama M, Kobayashi S, Shingu K, Nagashima H, Nagamine K, Kasuga Y, Kato R, Kameko F, Amano J. Solitary brain metastasis from papillary thyroid carcinoma in a patient with depression: report of a case. Surg Today. 2000;30(9):827-30. doi: 10.1007/s005950070066. PMID: 11039712. |

**SUPPLEMENTARY FIGURE 1**


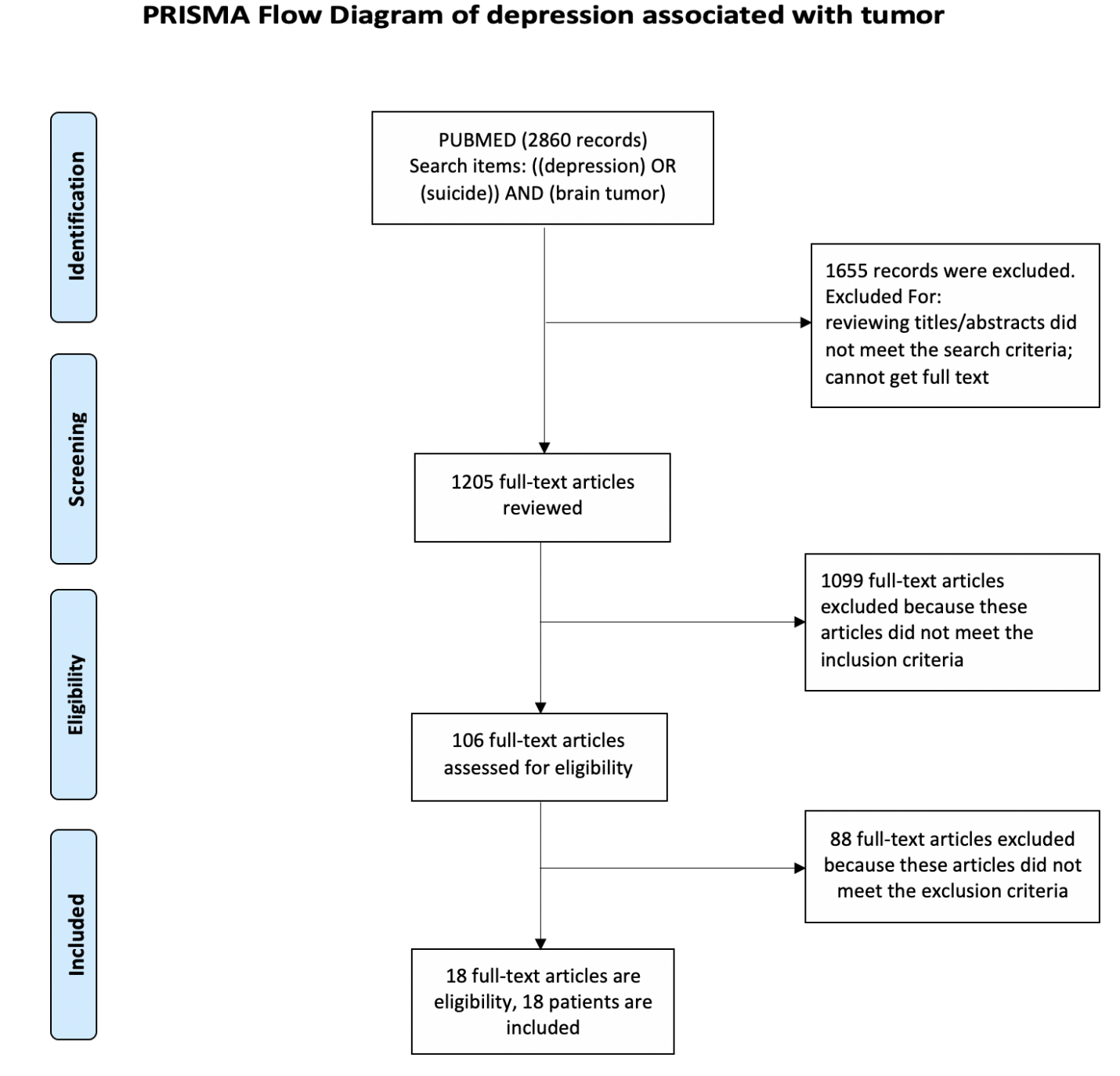


**Supplementary Figure 1:** Work flow for identifying cases of depression-causing tumors from the literature. A PubMed search for articles describing human subjects written in English with the search terms: ((depression) OR (suicide)) AND (brain tumor) most recently was performed. In total, 2860 articles matched initial search criteria. Of these, 1655 articles were excluded after reviewing the title. 1205 abstracts were reviewed and 1099 were rejected as they did not meet the inclusion criteria. The left 106 articles for full-text review with exclusion criteria. In total, 18 cases were included described in 18 articles.

**SUPPLEMENTARY FIGURE 2**


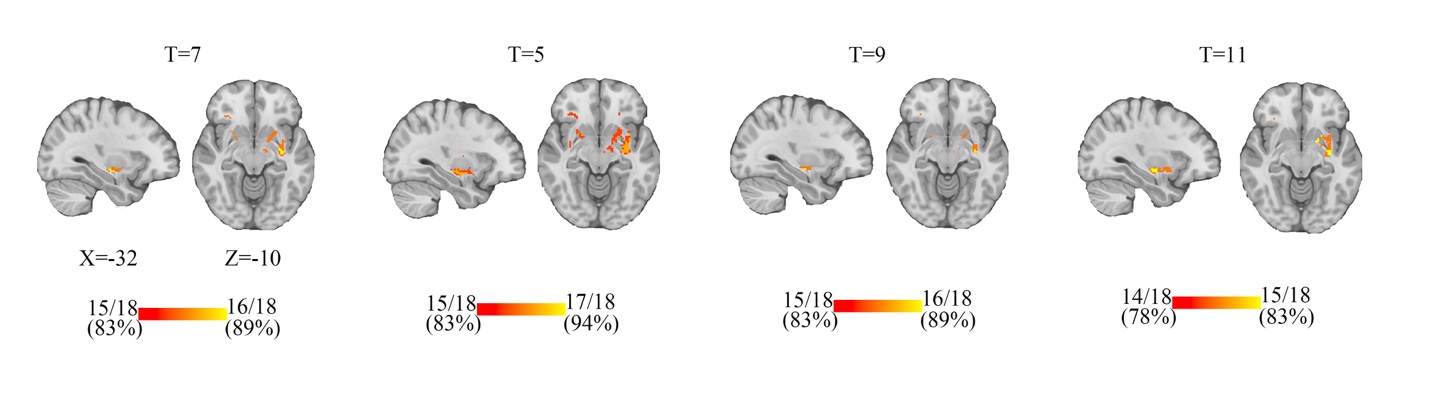


**Supplementary Figure 2:** Lesion network mapping of findings are independent of threshold.Regions of positive hub locates in the left striatum, which are similar across different network thresholds.
